# Postreproductive killer whale grandmothers improve the survival of their grandoffspring

**DOI:** 10.1073/pnas.1903844116

**Published:** 2019-12-09

**Authors:** Stuart Nattrass, Darren P. Croft, Samuel Ellis, Michael A. Cant, Michael N. Weiss, Brianna M. Wright, Eva Stredulinsky, Thomas Doniol-Valcroze, John K. B. Ford, Kenneth C. Balcomb, Daniel W. Franks

**Affiliations:** ^a^Department of Biology, The University of York, York YO10 5DD, United Kingdom;; ^b^Department of Computer Science, The University of York, York YO10 5DD, United Kingdom;; ^c^Centre for Research in Animal Behaviour, College of Life and Environmental Sciences, University of Exeter, Exeter EX4 4PY, United Kingdom;; ^d^Centre for Ecology and Conservation, University of Exeter in Cornwall, Penryn TR10 9FE, United Kingdom;; ^e^Center for Whale Research, Friday Harbor, WA 98250;; ^f^Pacific Biological Station, Fisheries and Oceans Canada, Nanaimo, BC V9T 6N7, Canada

**Keywords:** menopause, grandmother effect, grandmothering, postreproductive life span, killer whales

## Abstract

Understanding why females of some mammalian species cease ovulation prior to the end of life is a long-standing interdisciplinary and evolutionary challenge. In humans and some species of toothed whales, females can live for decades after stopping reproduction. This unusual life history trait is thought to have evolved, in part, due to the inclusive fitness benefits that postreproductive females gain by helping kin. In humans, grandmothers gain inclusive fitness benefits by increasing their number of surviving grandoffspring, referred to as the grandmother effect. Among toothed whales, the grandmother effect has not been rigorously tested. Here, we test for the grandmother effect in killer whales, by quantifying grandoffspring survival with living or recently deceased reproductive and postreproductive grandmothers, and show that postreproductive grandmothers provide significant survival benefits to their grandoffspring above that provided by reproductive grandmothers. This provides evidence of the grandmother effect in a nonhuman menopausal species. By stopping reproduction, grandmothers avoid reproductive conflict with their daughters, and offer increased benefits to their grandoffspring. The benefits postreproductive grandmothers provide to their grandoffspring are shown to be most important in difficult times where the salmon abundance is low to moderate. The postreproductive grandmother effect we report, together with the known costs of late-life reproduction in killer whales, can help explain the long postreproductive life spans of resident killer whales.

## Significance

Why humans and some species of whales go through menopause remains an evolutionary puzzle. In humans, postreproductive females gain genetic benefits by helping family members—particularly increasing their number of surviving grandoffspring. The extent to which these grandmother benefits are important in the evolution of menopause in whales remains unclear. Here, we test the grandmother effect in resident killer whales, where females can live for decades after their last reproductive event. We show that grandmothers increase the survival of their grandoffspring, and these effects are greatest when grandmothers are no longer reproducing. These findings can help explain why killer whales have evolved the longest postreproductive life span of all nonhuman animals.

Many mammals exhibit reproductive senescence, where fecundity declines with age (1). This reproductive senescence is typically aligned with somatic senescence—with both reproduction and survival gradually decreasing together with age (2, 3). In contrast, reproductive senescence is unusually accelerated relative to somatic senescence in humans (*Homo sapiens*), short-finned pilot whales (*Globicephala macrorhynchus*), belugas (*Delphinapterus leucas*), narwhals (*Monodon monoceros*), and resident-ecotype killer whales (*Orcinus orca*; hereafter killer whales) (4). This manifests as a prolonged postreproductive life span: a long period of a female’s life after reproduction where she can no longer reproduce (5). For example, approximately 3-quarters of women that survive into adulthood in hunter-gatherer societies do not give birth after 45 y, and they can expect to live into their mid-60s, on average (6, 7). Similarly, female killer whales also have extremely long postreproductive life spans; they stop reproducing in their late 30s and early 40s (4, 8) but can continue to live for decades thereafter (4, 5, 9); 64% of female killer whales that survive to the age of 10 y (approximately the point of sexual maturity) will live to be postreproductive (survive to at least age 45 y), at which point they will then have an expected postreproductive life span of 15.78 y.

Understanding why the female postreproductive life span has evolved in humans and some species of toothed whales has been a challenge for evolutionary biology. Research on humans suggests that the postreproductive life span has evolved, in part, due to the inclusive fitness benefits postreproductive females can gain by helping their kin (the grandmother hypothesis; refs. 10–12). In particular, the grandmother effect predicts that postreproductive grandmothers increase their inclusive fitness by supporting grandoffspring that are dependent on provisioned food for some time following weaning (13–15). There is substantial support for the grandmother effect across a range of human societies, including modern hunter-gatherer societies and preindustrial populations, which show that postreproductive grandmothers increase the survival of their grandoffspring, thus increasing their own inclusive fitness (10, 11, 14, 16–19). Although there is evidence for grandmother benefits in animals such as elephants (20, 21), there is no evidence for a postreproductive grandmother effect in nonhuman animals that have a prolonged female postreproductive life span.

For postreproductive females to be able to gain inclusive fitness benefits, they need both the opportunity to interact with grandoffspring and also a direct mechanism by which they can increase the survival of their kin. In killer whales—which are the best-studied species of toothed whales that exhibit a prolonged postreproductive life span—offspring do not disperse away from their mother (22, 23). This results in a close-knit family-based society, where grandmothers regularly group with both their offspring and their maternal grandoffspring. Previous research on killer whales has demonstrated a mother effect, with mothers increasing the survival of their weaned offspring (24). This effect is particularly strong for male offspring, but is irrespective of whether the mother is reproductive or postreproductive (24). In addition to supporting offspring to independence, postreproductive females might support grandoffspring directly by cooperative foraging and food sharing (25) or sharing ecological knowledge (26). This presents the clear potential for selection for helping grandoffspring in killer whales. Here we test the grandmother effect in killer whales by examining the survival of grandoffspring with living or recently deceased grandmothers. We also test whether postreproductive grandmothers support grandoffspring better than reproductive grandmothers. We control for the mother effect and for resource abundance in testing these hypotheses. Finally, postreproductive grandmothers can gain inclusive fitness benefits by reducing the interbirth interval of their daughters (18), and thus increasing their daughter’s lifetime reproductive success; thus we also examine the effect of mothers on the interbirth interval of their daughters.

## Methods

### Study Populations.

Demographic records were collected annually using photographic censuses for 2 resident killer whale populations: the southern (1976–2016) and northern (1973–2016) populations in the inshore coastal waters of Washington State and British Columbia, Canada (see refs. 9 and 27 for details). Resident killer whales are typically observed between May and November, when the animals frequent inshore waters. Individuals were identified by their unique fin shapes, saddle patches, and the presence of any nicks or scratches, and were sexed using distinctive pigmentation patterns around the genital slits and, in adults, differences in fin size. Genealogical relationships were inferred from long-term observations of social organization, and mothers were identified by their repeated association with young calves.

The data for each individual consisted of a year of birth, a year of death, and the identification (ID) of their mother when known. From this, we calculated age at death for all individuals, and maternal grandmother ID for those individuals whose mother had a known mother as well. Anonymized data can be accessed on OSF through the following link: https://bit.ly/2n5cBHU (28). Maternal grandmothers, and not paternal grandmothers, were assessed because there is no dispersal and thus paternal grandoffspring are raised outside of the group. For grandmothers born prior to the start of the annual censuses, we assigned estimated birth years based on birth histories and the ages of their offspring. We filtered the data (*n* = 726 individuals) to include only individuals with known maternal grandmothers, giving a sample size of 378 individuals (92 males, 76, females and 210 individuals of unknown sex who died before reproductive maturity). As there is no dispersal from either population, mortality was recorded if an individual’s matriline was observed in the population within a given year but the individual did not appear.

Annual indices of Chinook salmon (*Oncorhynchus tshawytscha*) catch from test fisheries were used as a measure for region-wide salmon abundance within each year in the Pacific Northwest (e.g., ref. 26). The abundance in each year was calculated as the mean salmon abundance from 3 different sites, covering the entire key habitat range of the resident killer whale populations: southeast Alaska, northern British Columbia, and the west coast of Vancouver Island. This salmon index comes from the Chinook Technical Committee abundance indices, which are based on the output of the Pacific Salmon Commission Chinook model, and aggregated from 3 aggregate abundance-based management fisheries areas (29) and is scaled such that an index of 1 is the mean abundance for the period 1979–1982 (see appendix G in ref. 30). These data were available from 1979 to 2015. Individuals observed before 1979 were therefore left-censored, leaving a dataset of 4,578 whale-years lived across the 378 individuals. Individuals who died within the same year as their birth were assigned an age at death of 0.01 (see below for details).

### Survival Model with Time-Dependent Effects.

We used an extended Cox proportional hazards model to examine the consequences of a grandmother’s death on grandoffspring survival, which accounts for right-censoring of data for some individuals (in this case, right-censoring occurred when an individual was still alive at the end of the study). The model was fit with maximum likelihood, and variations of the model were examined using Akaike information criterion (AIC) model selection; these are detailed below and are presented in further detail in *SI Appendix*. All analysis was implemented in R 3.5.0 (31) using the “survival” package (32).

Individual sex was coded as 0 for females and 1 for males, and individuals of unknown sex were coded as 0.5 (which gives equivalent results to randomizing sex). When a focal individual (of any age) died in the same year as their mother/grandmother (and thus we do not know for certain who died first), death order was randomized with equal chance of either dying first. The models ran with annual time steps, and, if the focal individual was determined to have died after their mother/grandmother, the age at death was set to the age in the year of death +0.01. This arbitrary small number is an implementation requirement, due to the model working in units of years, which allows us to capture the fact that the individual dies after its mother/grandmother, and could take any value between 0 and 1 with the same result. Because we are correlating mother/grandmother death with (grand)offspring death, not adding this number would implicitly add the assumption that (grand)offspring always die first. The death order matters because we examine mortality within a period following the mother’s or grandmother’s death. For each model, the population was run through 10,000 randomizations of the death order—for individuals with unknown death order—and the median coefficient, *P* values, and likelihoods were calculated.

There is a known effect of mother death in killer whales; adult sons are more likely to die in the 2 y following the death of their mother than similar-aged males with living mothers (24). Thus, we therefore controlled for grandoffspring who recently lost their mother by accounting for the contribution of the mother to the survival of her offspring. Previous work has shown that offspring show an increased mortality following the death of their mother, especially when the offspring are at least 30 y old when their mother dies. However, on the filtered dataset used here—of those individuals with a known grandmother—there were no individuals with a mother who died when the offspring was over 30 y old. We therefore did not include offspring age at mother death as a covariate. The best-fitting model of mother effect on survival on our current, reduced dataset captures the key significant short-term effect of mother death on the death of sons (24), and thus this term was retained in all statistical models to control for the mother effect (Eq. **1** and *SI Appendix*).

Our grandmother analysis took a variety of forms. We assign individuals to 3 categories depending on the status of their grandmother: 1) those with living grandmothers, which we consider as the baseline scenario; 2) those with grandmothers who died within the previous 2 y; and 3) those with grandmothers who died prior to the last 2 y. We chose a period of 2 y because we may expect some delayed effects of grandmother death on grandoffspring mortality. Within the 2 y, the calf might get by without its grandmother for a limited time, but, after surviving this period, the calf might be able to adjust, in a number of ways, to not having its grandmother around (e.g., by changing its social connections and gaining support from other females). Note that changing the threshold to 3, 4, or 5 y following the death of the grandmother does not significantly impact results, with the best-fitting model’s AIC varying by less than 1 for these changes. As such, we report the results with a threshold of 2 y. We also allow the model (prior to model selection) to capture the possibility that any survival benefits from grandmothers may be sex-specific. Thus, we include a separate series of terms that only affect males. Potential differences between postreproductive and reproductive grandmothers were captured with a term reflecting whether a grandmother is postreproductive, which we conservatively defined as those over 45 y old, consistent with previous work (26).

To control for the effect of Chinook salmon abundance on mortality (33), salmon abundance was included as a time-dependent variable. An interaction between salmon abundance and grandmothering was also considered, and we fitted models including interaction effects of salmon with each of the grandmother terms in the final grandmothering survival model.

Cox proportional hazards models return 2 types of results. First, there are coefficients representing the contribution of a term (e.g., grandmother death) in a similar manner to those in linear models. Second, they return hazard ratios (HRs; the exponential of the coefficients), which are a factor by which the risk of death is multiplied. An HR of 1 indicates that there is no change in mortality between cases, whereas a value above 1 indicates an increased risk of death. Reported confidence intervals represent the range of parameters returned from the randomizations within the model.

### Interbirth Interval Model.

We used a generalized additive model to examine the consequences of a grandmother’s status on her daughters’ interbirth intervals. To test whether grandmothers decrease their daughters’ interbirth intervals, we regressed a number of covariates on each interbirth interval. Here we refer to “daughter”’ as the female giving birth to the 2 calves in the interval, and “grandmother” as the mother of that daughter (and thus grandmother to the calves in the interval). We control for the daughter’s age in the model by fitting it as a smooth term. This is defined as the daughter’s age in the year when she gives birth to the second calf in the interval. We also included the following as covariates in the model: whether the first calf in the interval survived past the age of weaning [defined as 2 y (34)], whether the grandmother was alive 2 y into the birth interval (i.e., the time after which a surviving calf would be weaned), whether the grandmother was alive and postreproductive 2 y into the interval, and the mean salmon abundance during the interval. We note that—as with most long-term observational studies—the analysis is based on observed births, and there will be births that are unobserved due to calves dying very soon after birth (8). All full-model specifications can be found in *SI Appendix*, Table S12.

## Results

### Survival Model.

We considered a number of models with a variety of terms (*SI Appendix*) including a general grandmother effect, and an additional effect of the grandmother being postreproductive. AIC differences between models were small. Each of the models with a close match to the best model (AIC differences of <2) show a general grandmother effect, and we therefore focus on the model with the lowest AIC value (*SI Appendix*). Schoenfeld residuals of the best-fitting model indicate that the grandmother effect is consistent across all ages, meeting the proportional hazards requirements (χ^2^ = 1.71, *P* = 0.19), as does the grandmother age at death (χ^2^ = 1.28, *P* = 0.26) and salmon index (χ^2^ = 1.72, *P* = 0.19). The best-fitting survival model (AIC = 1,254.1) for the effect of grandmother status on grandoffspring survival gives the mortality hazard of an individual at age t (h(t)) by

$$h\left(t\right)=h_{0}\left(t\right)exp\left\{2.9\;sMR+4.3GMR+0.4GMo45-2.8\left(slm\times GMR\right)\right\},$$ [1]

where $h_{0}\left(t\right)$ is the baseline hazard at age *t*; *sMR* = sex when the focal individual’s mother has died in the last 2 y; GMR=1 when the focal individual’s grandmother has died in the last 2 y; GMo45=1 when the grandmother has died, and was older than 45 at the time of her death; slm is the value of the annual salmon index; and model coefficients are rounded to a single decimal place (see *SI Appendix* for more precise values).

**Fig. 1.**
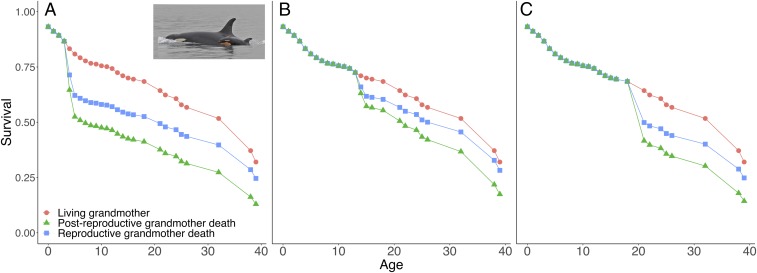
Example survival trajectories for (*A*) a 5-y-old whale, (*B*) a 15-y-old whale, and (*C*) a 20-y-old whale, when their grandmother is alive (red line/circles), when their reproductive grandmother has recently died (blue line/squares), and when their postreproductive grandmother has recently died (green line/triangles), where the salmon index is fixed at 1 across all years. Survival is derived from the best-fitting extended Cox proportional hazards model for grandoffspring experiencing their grandmother’s death at different ages (ages are in years). *Insert* shows grandmother J19 with her grandoffspring J51.

We find evidence for the grandmother effect in killer whales: The death of a grandmother reduces the survival of her male and female grandoffspring in the 2 y following her death. Grandoffspring whose maternal grandmother died within the last 2 y have a mortality HR 4.5 times higher than an individual with a living grandmother, when salmon is indexed at 1 (the mean abundance between years 1979 and 1982). Further, the model shows that those individuals who lose a postreproductive grandmother see their mortality increase above that of a reproductive grandmother by a factor of 1.5 (independent of salmon). Thus, an individual losing a postreproductive grandmother will see their mortality risk increase in total by a factor of 6.7 when the salmon index is 1. The AIC analysis shows that the *GMR* and *GMo*45 terms both have variable importance greater than 0.75. This means that the probability that these terms are in the Kullback–Leibler best model out of the candidate set is over 75%, demonstrating the importance of grandmothers and their postreproductive status in the model (*SI Appendix*, Table S2). Fig. 1 shows example survival trajectories for a 5-, 15-, and 20-y-old whale whose grandmother is alive (red line/circles), whose reproductive grandmother has recently died (blue line/squares), and whose postreproductive grandmother has recently died (green line/triangles). Adding the weaning status of grandoffspring (defining weaned as those over 2 y old) to the best-fitting model (AIC = 1,254.8; model 16) does not improve model fit, or show any indication that grandmother effects are specific to weaned or unweaned grandoffspring (*SI Appendix*, Tables S10 and S11).

When we consider interactions between the grandmother’s death and salmon abundance, the coefficient for the effect of the grandmother’s death decreases with a slope of −2.8 (95% CI: −6.5 to 1.5) per unit of salmon index. This means that an increase of salmon by the mean abundance (i.e., a doubling) reduces the coefficient of the grandmother effect by 2.8, while a decrease by the mean abundance increases the coefficient of the grandmother effect by 2.8. Thus, the grandmother effect operates when salmon index is lower than 1.536, which is above the mean salmon index. Seventy-eight of the 123 grandoffspring deaths occurred below this salmon threshold, despite survival being mitigated by grandmothers. When a grandmother is postreproductive, the threshold at which she provides benefits is raised further to a Chinook salmon index of 1.67.

Of the candidate models with ΔAIC < 2, all of the models included a mother effect (*sMR*) and grandmother effect (*GMR*). Six of these 7 models include the additional effect of a grandmother being postreproductive (*GMo*45), and only 1 includes the mother’s age (but also includes the *GMo*45 term). The impact of salmon as an additive term (impacting all whales equally) appears in 2 of the 7 models, but also appears 3 times in an interaction with *GMR*. Our findings differ from a previous study examining the survival benefits of grandmothers in the study populations (35). One difference between the studies is sample size: We were fortunate to have 7 y of additional data, adding 88 births, 38 deaths, and an additional 26 grandmothers becoming postreproductive. Importantly, our approach separates short- and long-term effects of a grandmother dying, so that the impact of a grandmother dying in the last few years is different from the impact of a grandmother dying decades ago. Additionally, the mother effect we include controls for differences in survival impacts between sons and daughters. This follows theory and previous evidence that mothers invest more in their sons than their daughters (24). We also control for salmon and account for censoring with a survival model.

We did not detect any sex-specific effects of grandmother loss on the survival of grandoffspring (there is no term for this in the final model, above). While this may be influenced by the large number of unsexed individuals in the dataset—when filtered for only individuals with a known grandmother—our analysis of the mother effect in this paper clearly demonstrates sex differences in survival. The presence of the term sMR in our model only affects male offspring, suggesting that we have enough power to detect sex effects.

### Interbirth Interval Model.

We considered a number of models with a variety of terms (*SI Appendix*). The best-fitting model (AIC = 961.8445; model 2; see *SI Appendix* for full details) did not include an effect of grandmothers on their daughters’ birth intervals,

IBI=4.21+s(DA)+1.4208 CA, [2]

where *IBI* is the length of the birth interval measured in years, *DA* is a smooth term for the daughter’s age at the end of the birth interval (i.e., at the time of the birth of the second calf in the interval), and *CA* is 1 if the first calf in the interval survives more than 2 y into the interval (i.e., does the calf survive to weaning); otherwise it is set to 0.

The smooth term *s*(*DA*) in this model was fitted with an estimated degree of freedom of 4.793. Two other models were, however, within ΔAIC < 2 of this best-fitting model. Those models included both terms from the best-fitting model (model 2; *SI Appendix* and Eq. **2**) as well as the effect of grandmothers on their daughters’ *IBI* (model 5; *SI Appendix*) and whether the grandmother was postreproductive (model 6; *SI Appendix*). In contrast to the hypothesized effects of grandmothers on their daughters’ *IBI*, these models actually suggest that grandmothers (and particularly postreproductive grandmothers) have a small effect of increasing the birth interval if they are alive at the time of weaning for the first calf (*SI Appendix*, Table S12).

## Discussion

We have shown that grandmothers bestow a survival advantage on their grandoffspring (a grandmother effect), and the effect remains after controlling for the mother effect. This is particularly the case when grandmothers are postreproductive, and thus provides evidence supporting the grandmother hypothesis in a nonhuman menopausal species. A key challenge in explaining the evolution of menopause across species is not just quantifying the benefits provided by postreproductive grandmothers—which explains why they live so long (36)—but also explaining why they do not continue reproducing. Here we have shown evidence that grandmother killer whales provide support to their grandoffspring, and that this is especially the case when the grandmothers are postreproductive. By stopping reproduction, postreproductive grandmothers not only avoid reproductive conflict with their daughters (23) but also offer increased benefits to their grandoffspring above that provided by reproductive grandmothers. Our data suggest that breeding grandmothers are not able to provide the same level of support as postreproductive grandmothers, and thus the evolution of reproductive termination increases a grandmother’s capacity to help. There are a number of potential mechanisms that may explain this finding. For example, it is possible that, when grandmothers are supporting their own calves, their movement and activity patterns are constrained and they are not able to act as leaders in the same way as postreproductive females (23). Moreover, grandmothers with their own calves will require more food for lactation and thus are perhaps less likely to share food with other group members. Further observational studies are needed to study the behavioral interactions between grandmothers and grandoffspring in resident killer whales.

We do not find support for the hypothesis that grandmothers (either reproductive or postreproductive) reduce the interbirth interval of their daughters, with the best-fitting model not including this effect and other models demonstrating a postreproductive grandmother’s presence being correlated with a small increase in the interbirth intervals of her daughters. We conjecture that this is because the grandmother would increase the survival of the first calf through the birth interval and thus impact her daughter in slightly delaying the birth of her second calf.

Our results further highlight the key role that postreproductive grandmothers play in killer whale societies in mitigating the impact of Chinook salmon abundance on the mortality risk of their grandoffspring. The impact of losing a postreproductive grandmother is highest in years with low and moderate salmon abundance. Killer whales forage selectively for Chinook salmon (30), and abundance of this prey species is known to have a strong negative correlation with killer whale mortality (33) and reproduction (8). Previous research has shown that postreproductive female killer whales act as repositories for ecological knowledge and that they provide an important leadership role for the group when foraging in salmon grounds (26). The importance of this leadership role in years of low Chinook salmon abundance may explain why the cost of losing a grandmother is slightly higher in years of low salmon abundance. As salmon populations continue decline, grandmothers are likely to increase further in importance for these killer whale populations (37, 38).

Consistent with previous findings—that postreproductive grandmothers confer benefits to their grandoffspring through a mechanism of leadership of the entire matriline (26)—we found no sex-specific effects of postreproductive grandmother loss on the survival of grandoffspring. This may be because the benefits of leadership around salmon foraging grounds cannot be directed toward specific kin, and both sexes of grandoffspring would be expected to benefit equally from leadership by their grandmother. This is in contrast to food sharing by mothers, which can be directed at specific individuals (such as males) within the group (25). There is no evidence that certain matrilines are more vulnerable than others (39), and so grandmothers are likely to be important across matrilines.

Outside of humans, menopause has only evolved in a small number of toothed whales (4, 5), and the long-term individual-based demographic data on resident killer whales provide a rare opportunity to test theoretical models on the evolution of menopause. In support of the grandmother hypothesis, we have shown that grandmothers increase the survival of their grandoffspring, and that postreproductive grandmothers are more effective helpers than grandmothers that continue to reproduce. These benefits to grandoffspring are necessary to explain why females have evolved to live long lives after they have terminated reproduction. Benefits alone, however, cannot explain why females terminate reproduction midway through life. Indeed, in other long-lived species that live in close-knit family groups, such as elephants, grandmothers provide benefits to grandoffspring while continuing to reproduce until the end of their long lives (20, 21). Examples such as this demonstrate that costs of continued reproduction are needed to explain why reproduction is terminated before the end of life (22). In killer whales and humans, intergenerational reproductive conflict has been found to provide such a cost, and thus select for early reproductive cessation. In killer whales, when mothers and daughters cobreed, the calves of mothers from older generation have significantly higher mortality (19, 23). Thus, for a complete understanding of the evolution of menopause, we need to move away from testing discrete hypotheses (e.g., mother, grandmother, reproductive conflict hypotheses) and instead take an integrated approach which considers both the fitness benefits of late-life helping to recipients (such as the grandmother effect reported here) and also the costs of late-life reproduction to breeders and other local group members (23). Only with this integrated approach can we fully explain why killer whales have evolved one of the longest postreproductive life spans recorded for all nonhuman animals.

## Supplementary Material

Supplementary File
