# Supplementary material for: Postreproductive killer whale grandmothers improve the survival of their grandoffspring
Source: Proc Natl Acad Sci U S A. 2019 Dec 9;116(52):26669–73. doi: 10.1073/pnas.1903844116 (PMC6936675; doi:10.1073/pnas.1903844116)
Supplement: Supplementary File [file pnas.1903844116.sapp.pdf]

Supplementary Information for

Post-reproductive Killer Whale Grandmothers Improve the Survival of their Grandoffspring

Stuart Nattrass, Darren Croft P., Samuel Ellis, Michael A. Cant, Michael N. Weiss, Brianna Wright, Eva Stredulinsky, Thomas Doniol-Valcroze, John K.B. Ford, Kenneth C. Balcomb, Daniel W. Franks

Corresponding author: Daniel Franks

Email: daniel.franks@york.ac.uk

This PDF file includes:

Supplementary text

Tables S1 to S12

Data Description

Supplementary information

Parameter list for coefficients used in the model comparisons:

sex = 1 if individual is male, 0 if female, and 0.5 if unknown

MR = 1 if mother died in last 2 years

ML = 1 if mother died prior to last 2 years

sMA = *sex* if mother is living

sMR = *sex* if MR=1

sML = *sex* if ML=1

gmA = 1 if grandmother is living

gmR = 1 if grandmother died in last 2 years

gmL = 1 if grandmother died prior to last 2 years

sgmA = *sex* if *gmA* = 1

sgmR = *sex* if *gmR* = 1

sgmL = *sex* if *gmL*= 1

gmo45 =1 if the grandmother has died and was aged over 45 (post-reproductive) at death

slm = observed salmon abundance in given year

mageAlive = current age of the focal individuals mother if she is alive, 0 otherwise

Each binary parameter is set to zero if their respective conditions are not met.

Model Selection

Each table 3-6 presents the median hazard coefficients of the variables over 10,000 randomisations of the death order. The final column represents our model selection criteria, where the lowest AIC is considered the most parsimonious model. The best model is highlighted in bold. Mother effect models were used to determine which mothering terms were included in the models, with the terms from the best mother model being carried over.

All statistical models were compared via AIC. We calculate ΔAIC for each of *R* models by subtracting the minimum AIC value from all AICs, and then calculate model weights (w) for each model *i* as

$$w_i = \frac{\exp\left(-0.5 \cdot \Delta\mathrm{AIC}_i\right)}{\sum_{r=1}^{R} \exp\left(-0.5 \cdot \Delta\mathrm{AIC}_r\right)}$$

This quantity is often interpreted as the probability that a given model is the K-L best model out of the candidate set (table 1).

Table S1. Summary of models and selection criteria. All models with ΔAIC < 2 are in bold

Model	Terms	AIC	ΔAIC	w
1	MR + ML + sMA + sMR + sML	1263.629	9.569495	0.001598
2	sMA + sMR + sML	1260.415	6.355493	0.007972
3	sMR	1258.323	4.263298	0.022692
4	gmA	1272.207	18.1471	2.19E-05
5	MR + ML + sMA + sMR + sML + mageA	1265.129	11.06929	0.000755
6	sMA + sMR + sML + mageA	1262.144	8.084595	0.003358
7	sMR + mageA	1260.102	6.04215	0.009324
8	sMR + gmR + gmL + sgmA + sgmR + sgmL + slm	1259.384	5.324206	0.013351
9	sMR + gmR + gmL + sgmA + sgmR + sgmL + gmo45 + slm	1260.588	6.528728	0.00731
**10**	**sMR + gmR + gmo45**	**1254.824**	**0.764295**	**0.13052**
**11**	**sMR + gmR + gmo45 + slm**	**1255.435**	**1.375307**	**0.096161**
12	sMR + gmR + slm	1256.759	2.699272	0.049602
**13**	**sMR + gmR**	**1255.882**	**1.821992**	**0.076913**
14	sMR + gmR + gmo45 + slm + gmR:slm + gmo45:slm	1257.172	3.112457	0.040344
**15**	**sMR + gmR + gmo45 + slm + gmR:slm**	**1255.353**	**1.293509**	**0.100175**
**16**	**sMR + gmR + gmo45 + gmR:slm**	**1254.06**	**0**	**0.191267**
17	sMR + gmR + gmo45 + slm + gmo45:slm	1257.148	3.088062	0.040839
**18**	**sMR + gmR + gmo45 + gmo45:slm**	**1255.42**	**1.360516**	**0.096874**
**19**	**sMR + gmR + gmo45 + gmR:slm + mageA**	**1256.055**	**1.995649**	**0.070517**

20	sMR + gmR + gmR:slm + mageA	1257.169	3.109427	0.040405

A term's model-averaged coefficient $\bar{\beta}$ is then

$$\bar{\beta} = \sum_{i=1}^{R} w_i \hat{\beta}_i$$

Where $\hat{\beta}$ is the estimated coefficient of the term in model *i*, which is 0 when the term is not included in the model.

Similarly, a term's variable importance is simply the sum of the weights of the models in which that term is included (table 2).

Table S2. Model averaged coefficients and variable importance for all terms. Terms with variable importance > 0.9 are in bold.

Term	Model Averaged Coefficient	Variable Importance
motherrecent	0.001574	0.002353
motherlong	0.000934	0.002353
sexmotheralive	0.004516	0.013683
**sexmotherrecent**	**2.884683**	**0.999978**
sexmotherlong	0.003597	0.013683
motheragealive	8.53E-05	0.124359
gmalive	-8.66E-06	2.19E-05
**gmrecent**	**2.128937**	**0.954278**
gmlong	0.000734	0.020661
sexgmalive	0.001895	0.020661
sexgmrecent	0.008289	0.020661
sexgmlong	0.010244	0.020661
gmover45	0.37338	0.774007
salmon	-0.06953	0.347782
gmRslm	-1.11666	0.442708
gmo45slm	-0.05812	0.178058

Mother effect model selection

Model	MR	ML	sMA	sMR	sML	AIC
1	0.587 (-1.659: 2.903) P=0.594	0.276 (-0.789: 1.341) P=0.612	0.363 (-0.135: 0.861) P=0.153	2.503 (-0.327: 5.174) P=0.081	0.056 (-1.294: 1.409) P=0.935	1263.629
2			0.327 (-0.149: 0.805) P=0.179	3.079 (2.017: 4.155) P=1.0e-8	0.285 (-0.735: 1.305) P=0.584	1260.415
**3**				2.893 (1.831: 3.927) P=8.4e-8		1258.323

Table S3: Statistics for fitted models with only the mother survival terms, and sex terms.

Grandmother alive/dead only model

Model	gmA	AIC
4	-0.395 (-0.775: -0.015) P=0.042	1272.207

Table S4: Statistics for the fitted model with only the grandmother alive/dead survival term.

Mother effect model selection including mageAlive

Model	MR	ML	sMA	sMR	sML	mageAlive	AIC
5	0.841 (-1.507: 3.214) P=0.493	0.653 (-0.737: 2.046) P=0.358	0.347 (-0.149: 0.846) P=0.170	2.587 (-0.296: 5.313) P=0.077	0.045 (-1.316: 1.407) P=0.948	0.009 (-0.013: 0.032) P=0.413	1265.129
6			0.316 (-0.171: 0.804) P=0.204	3.150 (1.955: 4.357) P=2.6e-7	0.358 (-0.830: 1.546) P=0.555	0.002 (-0.154: 0.020) P=0.801	1262.144

7				2.968<br>(1.839:<br>4.100)<br>P=1.8e-7		0.002<br>(-0.012:<br>0.017)<br>P=0.763	1260.102

Table S5: Statistics for fitted models with only the mother survival terms, and sex terms, but with current mother age added to each model from table S3.

Grandmother model selection

Model	sMR	gmR	gmL	$sgmA$	$sgmR$	$sgmL$	$gmo45$	slm	AIC
8	2.622<br>(1.561:<br>3.683)<br>P=9.7e-7	0.813<br>(-0.625:<br>2.254)<br>P=0.268	0.082<br>(-0.589:<br>0.752)<br>P=0.811	0.091<br>(-0.646:<br>0.826)<br>P=0.809	0.408<br>(-1.413:<br>2.262)<br>P=0.654	0.503<br>(-0.118:<br>1.126)<br>P=0.113		-0.233<br>(-0.665:<br>0.200)<br>P=0.292	1259.384
9	2.668<br>(1.597:<br>3.748)<br>P=9.7e-7	0.652<br>(-0.852:<br>2.149)<br>P=0.395	-0.049<br>(-0.790:<br>0.690)<br>P=0.892	0.094<br>(-0.644:<br>0.830)<br>P=0.804	0.389<br>(-1.448:<br>2.261)<br>P=0.673	0.482<br>(-0.139:<br>1.104)<br>P=0.128	0.253<br>(-0.288:<br>0.795)<br>P=0.359	-0.232<br>(-0.665:<br>0.201)<br>P=0.294	1260.588
10	2.926<br>(1.876:<br>3.974)<br>P=4.5e-8	0.718<br>(-0.135:<br>1.570)<br>P=0.099					0.386<br>(-0.019:<br>0.792)<br>P=0.062		1254.824
11	2.863<br>(1.810:<br>3.915)<br>P=1.0e-7	0.688<br>(-0.169:<br>1.544)<br>P=0.116					0.384<br>(-0.022:<br>0.790)<br>P=0.063	-0.237<br>(-0.672:<br>0.199)<br>P=0.287	1255.435
12	2.823	0.887						-0.238	1256.759

	(1.777: 3.878) P=1.5e-7	(0.058: 1.716) P=0.036						(-0.673: 0.197) P=0.283	
13	2.899 (1.853: 3.940) P=5.3e-8	0.924 (0.097: 1.750) P=0.029							1255.882

Table S6: Statistics for fitted models with mother, grandmother, sex interactions, and additive salmon terms.

Interaction between grandmothering and salmon abundance

Model	*sMR*	*gmR*	*Gmo45*	*slm*	*gmR*slm*	*Gmo45*slm*	*AIC*
14	2.859 (1.802: 3.922) P=1.3e-7	1.477 (-1.683: 4.858) P=0.369	0.648 (-0.703: 2.001) P=0.347	-0.129 (-0.671: 0.414) P=0.643	-0.578 (-3.327: 1.997) P=0.671	-0.202 (-10108: 0.704) P=0.662	1257.172
15	2.853 (1.795: 3.912) P=1.3e-7	3.853 (-1.129: 8.169) P=0.107	0.369 (-0.041: 0.778) P=0.078	-0.193 (-0.632: 0.246_ P=0.388	-2.496 (-6.258: 0.246) P=0.194		1255.353
**16**	**2.898 (1.842: 3.955) P=7.6e-8**	**4.318 (-0.731: 8.544) P=0.086**	**0.368 (-0.041: 0.778) P=0.078**		**-2.811 (-6.542: 1.533) P=0.152**		**1254.06**
17	2.868 (1.816: 3.925) P=1.0e-7	0.657 (-0.202: 1.518) P=0.134	0.767 (-0.570: 2.103) P=0.261	-0.137 (-0.681: 0.406) P=0.621		-0.267 (-1.166: 0.632) P=0.561	1257.148
18	2.891 (1.843: 3.945) P=7.3e-8	0.657 (-0.202: 1.517) P=0.134	0.961 (-0.123: 2.046) P=0.082			-0.403 (-1.124: 0.318) 0.274	1255.42

Table S7: Statistics for fitted models that include an interaction between grandmother death and salmon index

Comparison of mother age and post-reproductive grandmother effects in best fitting model

Model	sMR	gmR	Gmo45	gmR:slm	mageAlive	AIC
19	2.894 (1.750: 4.037) P=7.8e-7	4.167 (-0.786: 8.432) P=0.086	0.372 (-0.046: 0.789) P=0.081	-2.704 (-6.441: 1.526) P=0.156	-0.0004 (-0.015: 0.014) P=0.917	1256.055
20	2.940 (1.793: 4.086) P=5.4e-7	4.519 (-0.375: 8.808) P=0.065		-2.847 (-6.618: 1.391) P=0.139	0.002 (-0.013: 0.017) P=0.789	1257.169

Table S8: Statistics for models comparing the best fitting model from Table S7 to that same model including current mother age, and replacing grandmother age at death with current mother age

Baseline hazard results for model 16

Age	survival
0	1
0.01	0.9317914
1	0.9109458
2	0.8922604
3	0.8662591
4	0.8325562
5	0.8086164
6	0.7917492
7	0.7774825
8	0.7666257
9	0.7630136
10	0.7554141
11	0.7515013
12	0.7428485
13	0.7245344
14	0.7102096
15	0.7001025
16	0.6948398
17	0.6948398
18	0.6842934
19	0.6842934
20	0.6842934
21	0.6432206
22	0.6233646
23	0.6233646
24	0.6067848

25	0.5789901
26	0.5674077
27	0.5674077
28	0.5674077
29	0.5674077
30	0.5674077
31	0.5674077
32	0.5171197
33	0.5171197
34	0.5171197
35	0.5171197
36	0.5171197
37	0.5171197
38	0.3716110
39	0.3202017
40	0.3202017
41	0.3202017

Table S9: The baseline hazard for the best fitting model 16. This baseline is the case where both mother and grandmother remain alive for the duration

Data Information

Each row of data accounts for a period of an individual whale's life. This time period is generally a single year, except in cases where multiple family members die within the same year. In this case, the period of time covered by a row is assigned a duration of 0.01 years.

The data as presented contains the assumption that the focal individual dies last whenever there are multiple family members who die within the same year. The column p.rowexists is then used to facilitate randomisation of death order (see below)

Variable Details

- Id
  - Anonymised identifier for the individual whale
- X.mother
  - Anonymised identifier for the mother of the focal individual. Hereafter referred to as "the mother"
- Grandmother
  - Anonymised identifier for the focal individual's grandmother. Hereafter referred to as "the grandmother"
- Sex
  - 1 if male, 0 if female. Individuals of unknown sex are assigned a value of 0.5, equivalent to randomised sex

- Salmon
    - Salmon index value for the time period considered
- Motheralive
    - 1 if the mother is still alive during this time period, 0 otherwise
- Motherrecent
    - 1 if the mother has died within the previous 2 years, 0 otherwise
- Motherlong
    - 1 if the mother has died prior to the previous two years, 0 otherwise
- Gmalive
    - 1 if the grandmother is alive during this time period, 0 otherwise
- Gmrecent
    - 1 if the grandmother has died within the previous 2 years, 0 otherwise
- Gmlong
    - 1 if the grandmother has died prior to the previous two years, 0 otherwise
- Sexmotheralive
    - The values of columns “sex” and “motheralive” multipled
- Sexmotherrecent
    - The values of columns “sex” and “motherrecent” multipled
- Sexmotherlong
    - The values of columns “sex” and “motherlong” multipled
- Sexgmalive
    - The values of columns “sex” and “gmalive” multipled
- Sexgmrecent
    - The values of columns “sex” and “gmrecent” multipled
- Sexgmlong
    - The values of columns “sex” and “gmlong” multipled
- Gmover45
    - 1 if the grandmother has died and was aged 45 or over at the time of death, 0 otherwise
- gmRslm
    - The values of columns “gmrecent” and “salmon” multipled
- gmo45slm
    - The values of columns “gmover45” and “salmon” multipled
- Startage
    - Age of the focal individual at the beginning of the time period
- Stopage
    - Age of the focal individual at the end of the time period
- Event
    - 1 if the individual dies during the time period, 0 otherwise
- p.rowexists
    - This column is used to determine the randomisation of death orders. 0 if the row is included in the analysis regardless of randomisation. 0.5 if the row is dependent on death order. If this is the case and p.rowexists=0.5, then the row will be excluded from analysis with probability 0.5, and the event signified with be moved to the previous row. This switches the assumed death order from 1) ancestors, 2) focal individual to 1) focal individual, 2) ancestors

Survival Model for weaned individuals

We extend our main models by taking the best fitting model (AIC = 1254.06; model 16) and adding parameters that consider the grandmother effects only for weaning individuals (define as those aged 2 or over). Neither of these models improve model fit, or show any indication that grandmother effects are specific to weaned or unweaned grandoffspring.

Weaning model 1: death ~ Sexmotherrecent + GMrecent + GMover45 + GMrecent*salmon + GMrecent*weaned

AIC = 1256.0

Parameter	coeff	CI	P value
Sexmotherrecent	2.90	1.84 – 3.95	8.1e-8 *
Gmrecent	1.98	-1.06 – 5.25	0.20
Gmover45	0.37	-0.040 – 0.78	0.077
gmRslm	-1.17	-3.87 – 1.58	0.39
Gmrecent*weaned	0.15	-1.56 – 1.88	0.78

Table S10: Statistics for weaning model 1, which takes the best fitting previous model (model 16) and includes the weaning status of grand offspring.

Weaning model 2: death ~ Sexmotherrecent + GMrecent + GMover45 + GMrecent*salmon + Gmrecent*weaned + GMver45*weaned

AIC = 1256.1

Parameter	coeff	CI	P value
Sexmotherrecent	2.91	1.85 – 3.97	7.4e-8 *
Gmrecent	4.67	-0.44 – 9.07	0.067
Gmover45	-0.11	-0.97 – 0.76	0.80
gmRslm	-2.73	-6.58 – 1.53	0.17
Gmrecent*weaned	-0.25	-2.04 – 1.59	0.77
Gmover45*weaned	0.65	-0.33 – 1.64	0.19

Table S11: Statistics for weaning model 2, which takes the best fitting previous model (model 16) and includes the weaning status of grand offspring.

Interbirth Interval Model

We used a generalized additive model with a Gamma error structure and identity link function, to examine the consequences of a grandmother's alive/dead status on her daughters' interbirth interval by regressing a number of covariates on each birth interval length. We used AIC to select between a number of models capturing a range of covariates:

- birth.interval
  - The length, in years, of the birth interval.
- daughter.age
  - The age of the daughter at the end of the birth interval (i.e. her age in the year when the second calf in the interval is born).
- first.calf.alive.post.weaning
  - 1 if the first calf in the interval survives more than 2 years into the interval (i.e. has been weaned), otherwise it is set to 0.
- gmother.alive.end.first.calf.weaning
  - 1 if the grandmother (mother of the daughter whose interval is being examined) is alive more than two years into the interval (the time after which the first calf in the interval will, if alive, have been weaned), otherwise is it set to 0.
- gmother.alive.and.postreproductive.end.first.calf.weaning
  - 1 if gmother.alive.end.first.calf.weaning = 1 and the grandmother is over 45 two years into the interval (i.e. she is alive and postreproductive by the time the first calf has had time to be weaned), otherwise it is set to 0.
- salmon
  - The mean salmon abundance during the entire interval.

Proposed Models

Model 1 (AIC: 975.453): birth.interval ~ s(daughter.age)

Model 2 (AIC: 961.8445): birth.interval ~ s(daughter.age) + first.calf.alive.post.weaning

Model 3 (AIC: 976.4604): birth.interval ~ s(daughter.age) + gmother.alive.end.first.calf.weaning

Model 4 (AIC: 962.9218): birth.interval ~ s(daughter.age) + first.calf.alive.post.weaning + gmother.alive.end.first.calf.weaning

Model 5 (AIC: 962.27): birth.interval ~ s(daughter.age) + first.calf.alive.post.weaning + gmother.alive.end.first.calf.weaning + gmother.alive.and.postreproductive.end.first.calf.weaning

Model 6 (AIC: 963.9162): birth.interval ~ s(daughter.age) + first.calf.alive.post.weaning + gmother.alive.end.first.calf.weaning + gmother.alive.and.postreproductive.end.first.calf.weaning + salmon

Model	*(intercept)*	*s(daughter.age)*	*first.calf.alive.post.weaning*	*gmother.alive.end. first.calf.weaning*	*gmother.alive.and.postreproductive. end.first.calf.weaning*	*salmon*	*AIC*
1	5.4820	Edf=4.645 (P=1.14e-12)					975.453
**2**	**4.2152**	**Edf=4.793 (P=2.17e-09)**	**1.4208 (P=1.55e-05)**				**961.8445**
3	5.2444	Edf=4.596 (P=6.25e-12)		0.3466 (P=0.301)			976.4604

4	4.0161	Edf=4.766 (P=5.68e-09)	1.4085 (P=1.85e-05)	0.3077 (P=0.341)			962.9218
5	3.93881	Edf=4.791 (P=4.13e-06)	1.48613 (P=4.13e-06)	-0.03823 (P=0.9166)	0.57121 (P=0.0824)		962.27
6	3.67903	Edf=4.823 (P=6.44e-10)	1.49564 (P=3.83e-06)	-0.04632 (P=0.8991)	0.57577 (P=0.0806)	0.21004 (P=0.6325)	963.9162

Table S12: Statistics for the proposed inter birth interval models. Model 2 is the best fitting, although models 4 and 5 are within ΔAIC < 2 of model 2.
